# Chromatographic assay of recently approved co-formulation of Vonoprazan fumarate with low dose Aspirin: AGREE, Complex MoGAPI, and RGB 12-model assessments

**DOI:** 10.1186/s13065-024-01344-7

**Published:** 2024-11-16

**Authors:** Mona M. Abdel Moneim, Mohamed M. A. Hamdy

**Affiliations:** Department of Pharmaceutical Chemistry, Faculty of Pharmacy, Pharos University in Alexandria, Canal El Mahmoudia Street, Beside Green Plaza Complex 21648, Alexandria, Egypt

**Keywords:** Aspirin, Vonoprazan, HPLC, HPTLC, Green chromatography

## Abstract

Two simple, valid and green chromatographic based techniques are developed in the present work for first time to simultaneously analyze the recently approved combination of Aspirin (ASP) with the novel gastro-protective agent Vonoprazan (VON). First method is an HPLC-DAD “diode array detection”, where separation was successful using C18 (250 × 4.6 mm) column with isocratic elution of phosphate buffer-pH 6.8 and acetonitrile in ratio of 63:37 with detection at 230 nm. Second method is an HPTLC method on HPTLC silica plates using ethyl acetate: ethanol (75%): ammonia (5:5:0.05 v/v) mobile phase followed by densitometric scanning at 230 nm. The methods were applied successfully for analysis of VON and ASP mixture in laboratory-prepared tablets and the methods were validated in regards to linearity, precision, accuracy and selectivity. The proposed methods are assessed for their greenness and whiteness as well using the “Analytical GREEnness Metric Approach”, “Complementary Modified Green Analytical Procedure Index” and the new algorithm “RGB 12 model” (Red-Green-Blue) and proved the greenness and the sustainability of the methods in the routine assay of the newly marketed formulation.

**Supplementary Information:**

The online version contains supplementary material available at 10.1186/s13065-024-01344-7.

## Introduction

Acetylsalicylic acid, Aspirin, was first developed as a NSAID “non-steroidal anti-inflammatory drug” for managing fever, pain, and inflammation. However, Aspirin also irreversibly inhibits COX1, one of the two isozymes of Cyclooxygenase – COX, inhibiting the synthesis of platelet thromboxane A2, which promotes platelet aggregation. Thus, low doses (75–81 mg) of Aspirin became the key antiplatelet and antithrombotic drug used as prophylaxis for many cardiovascular diseases especially for high risk patients with history of myocardial infarction, coronary artery bypass graft surgery (CABG), or atrial fibrillation (AFib). Wide usage of Aspirin for protection against cardiac diseases, as well as its analgesic and antipyretic effect, makes this compound one of the most prescribed and used medications worldwide [1–3].

The cardioprotective benefits of low aspirin doses might be outweighed by its gastrointestinal complications on mucosa of the upper and lower gastrointestinal tract. However, those complications range from minor ones such as erosions to more serious ones as ulcers, and even could reach to death. The risk of those gastrointestinal side effects is also increased with old age (> 70), male sex, *H. pylori* infection, ulcer history and concomitant medication with other drugs such as corticosteroids or other antithrombotic agents [2–4].

Some key strategies have been adopted to minimize those aspirin related effects. These measurements include using other platelet inhibitor such as clopidogrel, co-administration of a gastro-protective agent with aspirin and eradication of *H. pylori* infections. Co-therapy with PPI (proton pump inhibitors) as gastro-protective agents is currently the most widely used strategy to reduce aspirin-related gastric complications [2, 3].

**Fig. 1:**
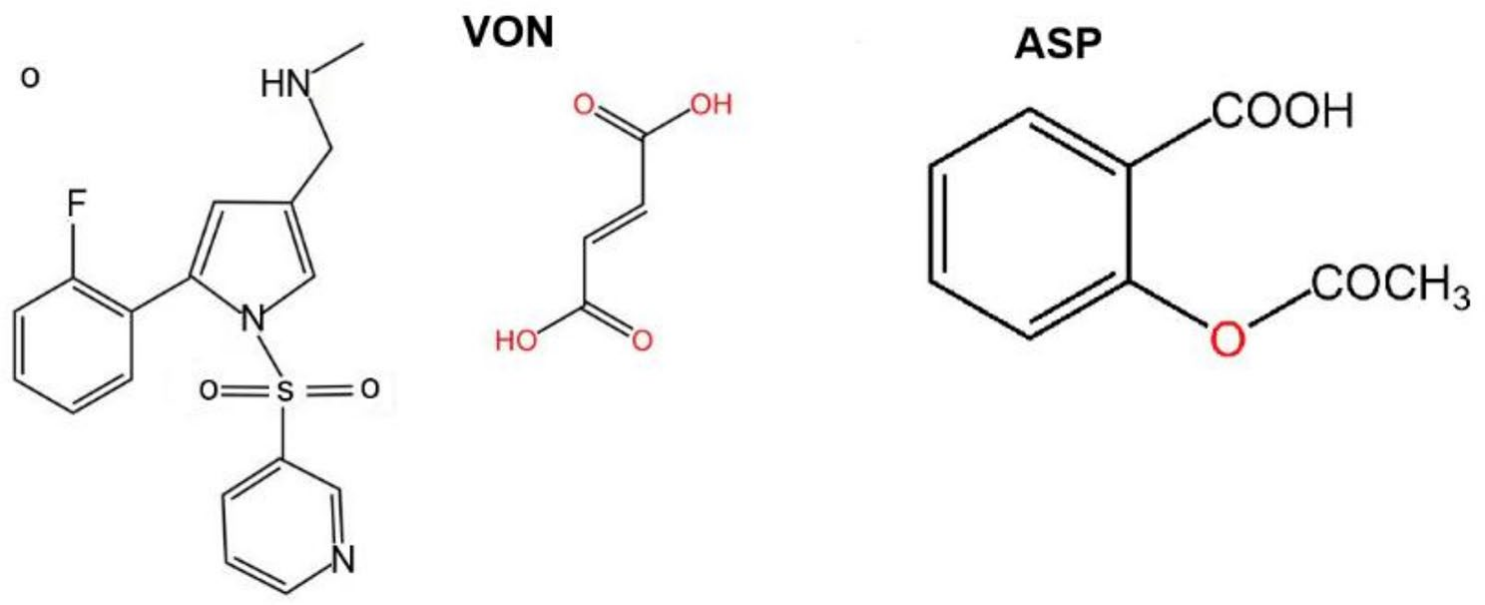
Chemical Structures of Vonoprazan Fumarate (VON) and Aspirin (ASP)

Meanwhile, “Takeda Pharmaceutical Company Limited” (Japan) developed Vonoprazan fumarate (1-(5-(2-fluorophenyl)-1-(pyridin-3-ylsulfonyl)-1 H-pyrrol-3-yl)-N-methyl methanamine mono fumarate) (Fig. 1) which is the first entity in a new gastro-protective class working as P-CAB (K^+^-competitive acid blockers). On February 2015, Vonoprazan was first approved in the Japanese market for management of gastroduodenal ulcers and reflux esophagitis. It is also combined with antibiotics for *H. pylori* infections treatment at doses of 10–20 mg [5–7].

Fixed dose drugs combination (FDCs) are necessary because they are advantageous over single therapy of multiple drugs in having better efficacy, lower side effects, high patient compliance, reported synergistic effect in some cases, and the most important factor of lower total therapeutic cost [8].

In this context, the two pharmaceutical companies “Otsuka” and “Takeda” launched Cabpirin^®^ which is combination tablets of Vonoprazan fumarate (VON) and Aspirin (ASP) in the ratio of 10 mg VON and 100 mg ASP to be taken once daily. By this innovative combination, which is approved by Japanese Ministry of Health, it is expected that patient adherence could be improved, and the recurrence of aspirin associated gastric and duodenal ulcers, may be reduced as well by the gastro-protective effect of VON. This new combination is considered a superior alternative to combination therapy of ASP with common PPIs.

The literature review shows no chromatographic method reported for this novel combination. Only one spectrophotometric assay [6] and another spectrofluorimetric one [9] had been reported so far. However, each drug separately had been previously analyzed by different analytical methods either alone or with other drugs in several matrices including HPLC [10, 11], UPLC and HPTLC [12], spectrophotometry [13, 14], spectrofluoimetry [15, 16], electrochemistry [17] and GC [18].

Chromatography is a widely used analytical technique in all laboratories due to its reproducibility, accuracy, low cost and highthroughput but there is no reported chromatographic methods to quantitatively analyse the mixture under study in their dosage form ratio. The current work proposes the first chromatographic assay of this mixture to be used for quality control analysis of its marketed dosage form. Since this combined binary mixture is expected to be commonly used in several countries in the near future due to its superiority in respect to patient adherence and its dual cardiac and gastric protective effects, it was important to ensure that the proposed methods for their assay in our current study are valid as well as green to be used routinely without breaching the current trend of green analysis and environmental sustainability. In that context, the proposed methods were validated, according to the ICH “International Council for Harmonization”, and also assessed for their greenness to assure the degree of their sustainability. Three different approaches were used for this purpose including RGB 12-model which combines the assessment of greenness as well validation of the methods under study in what called “Whiteness assessment”. Also using three different assessment approaches will highlight the differences between each assessment matrix where each one focuses on certain greenness values and perspectives [19].

## Experimental

### Chemicals and reagents

VON (Purity > = 98%), and ASP (Purity > = 99%) were obtained from Abcam (USA) and Medizen Pharmaceutical Ind. (Egypt), respectively. Acetonitrile HPLC-grade (Sigma-Aldrich Chemie GmbH, Switzerland), ortho-phosphoric acid and potassium dihydrogen orthophosphate (BDH Laboratory Suppliers, England) and double distilled water have been used. Analytical grade ethyl acetate, ammonia and ethanol (ElNasr Pharmaceutical Chemicals, Cairo, Egypt) were used for the HPTLC mobile phase.

Because Cabpirin^®^ tablets is only marketed in the Japanese market, laboratory-prepared tablet containing 10 and 100 mg of VON and ASP, respectively, per tablet was prepared with commonly used tablet excipients (starch, cellulose, Mg sterate, HPMC and silica) kindly gifted by Pharco Pharm. Co., Egypt.

### Instrumentation and chromatographic conditions

The HPLC separation of VON and ASP was performed using Agilent 1260 HPLC device equipped with Diode Array Detector (set at 230 nm for detection of both drugs) and Agilent Chem-Station Software. A reversed phase C18 column (250 × 4.6 mm, 5 μm) thermostated at 30^o^C has been used in this study with mobile phase of phosphate buffer of pH 6.8 (0.01 M potassium dihydrogen phosphate (1.36 g/L) in 1000 mL water, pH adjusted by 5 N sodium hydroxide solution) and acetonitrile in ratio of 63:37 with flow rate of 1 mL.min^− 1^.

For HPTLC assay, the used stationary phase for separation was 20 × 10 cm aluminum plates [silica gel-60 _(F254)_], from E. Merck, Germany. The sample (10 µL) was injected on the plate as 5 mm bands (5 mm apart) using a 100 µL Camag microsyringe & Linomat IV applicator. A mobile phase of ethyl acetate: ethanol (75%): ammonia (5:5:0.05) developed the plates in a Camag chamber (20 × 20 cm) after its saturation with the used mobile phase for at least 30 min followed by densitometric scanning using deuterium lamp and Camag scanner-III. Detection was also done at 230 nm after the spots dried on the plate. ((absorbance mode; deuterium lamp; 6 mm band width; 20 mm.s^− 1^ scanning speed; 5 × 0.45 mm slit dimensions).

### Methods

#### Standard stock solutions preparations

VON and ASP stock solutions were prepared by dissolving 20 (equiv. to 26.7 mg VON-fumarate) and 200 mg of each drug, respectively in 100 mL solvent. The solvent comprised of water and acetonitrile in ratio of 50:50 as previously reported for VON, HPLC single assay [10].

#### Calibration standards and synthetic mixtures

Separately, accurate volumes of VON (25–500 µL for HPLC and 0.5–10 mL for HPTLC) and ASP (5-500 µL for HPLC and 50 µL–5 mL for HPTLC) stock solution were transferred to two separate sets of 10-mL volumetric flasks and completed to volume with the same solvent to prepare calibration standards within ranges stated in Table 1. In addition, appropriate aliquots of each drug’s standard stock solution were added into another set of five 10-mL volumetric flasks and completed to mark as well with solvent to prepare synthetic mixtures in different ratios of both drugs as mentioned in Table 2. From each calibration standard and synthetic mixture prepared, triplicates HPLC injections (30 µL) were injected on the HPLC system and chromatographed using the optimized conditions previously mentioned. For the HPTLC work, triplicate (10 µL) injections were spotted as 5 mm bands on HPTLC plate and the plate was then developed ascendingly using the previously mentioned solvent.

#### Laboratory -prepared tablets

A portion of VON and ASP active ingredients equivalent to 10 and 100 mg, respectively, were mixed well with small amount of tablet fillers into a 50-mL volumetric flask and extracted using 20 mL solvent. After sonication for 10 min, dilution to volume by the same solvent and filtration was done. Appropriate volume of the prepared sample filtrate was then diluted into 10-mL volume to reach a concentration in the linearity range of each method.

## Results and discussion

Despite the fact that, the two concerned drugs in this research are assayed separately in previous literature, the literature lacks any chromatographic method for simultaneous assay of these two drugs in their binary mixtures. Since the combined dosage form of VON and ASP are recently launched and approved, there was an urgency to develop a chromatographic method to assay the two drugs simultaneously as the reported methods are valid to determine each drug individually and not in combination. The challenge in this work was to separate VON and ASP with reasonable retention times, symmetrical peaks and good resolution despite of their different physical and chemical properties. Moreover, the challenge was to separate them in the ratio of the tablet dosage forms, where the ratio of VON (10 mg) to ASP (100 mg) is 1:10. The proposed HPLC method can determine the two drugs in 10 min. run time and can be used to chromatographically assay the drugs individually or simultaneously. In addition, the HPTLC method offers the advantages of using relatively cheap instrumentation, low solvent consumption (where only 20 mL can develop a plate spotted with 18–20 samples each run), higher environmental protection and high throughput by running several samples simultaneously in the same run, which offers less time and cost per analysis. Meanwhile, the proposed chromatographic methods are considered more direct than the reported spectrophotometric and spectrofluorimetric techniques assay [6, 9] as they do not include any mathematical manipulation nor colorimetric reactions with numerous steps and reagents.

### Optimization of the chromatographic conditions

***HPLC method***: All chromatographic parameters have been validated including the stationary phase used where different columns: C8 (250 × 4.6 mm), C18 (150 × 4.6 mm) and C18 (250 × 4.6 mm) had been tried, but the best separation was done using the selected column.

**Fig. 2:**
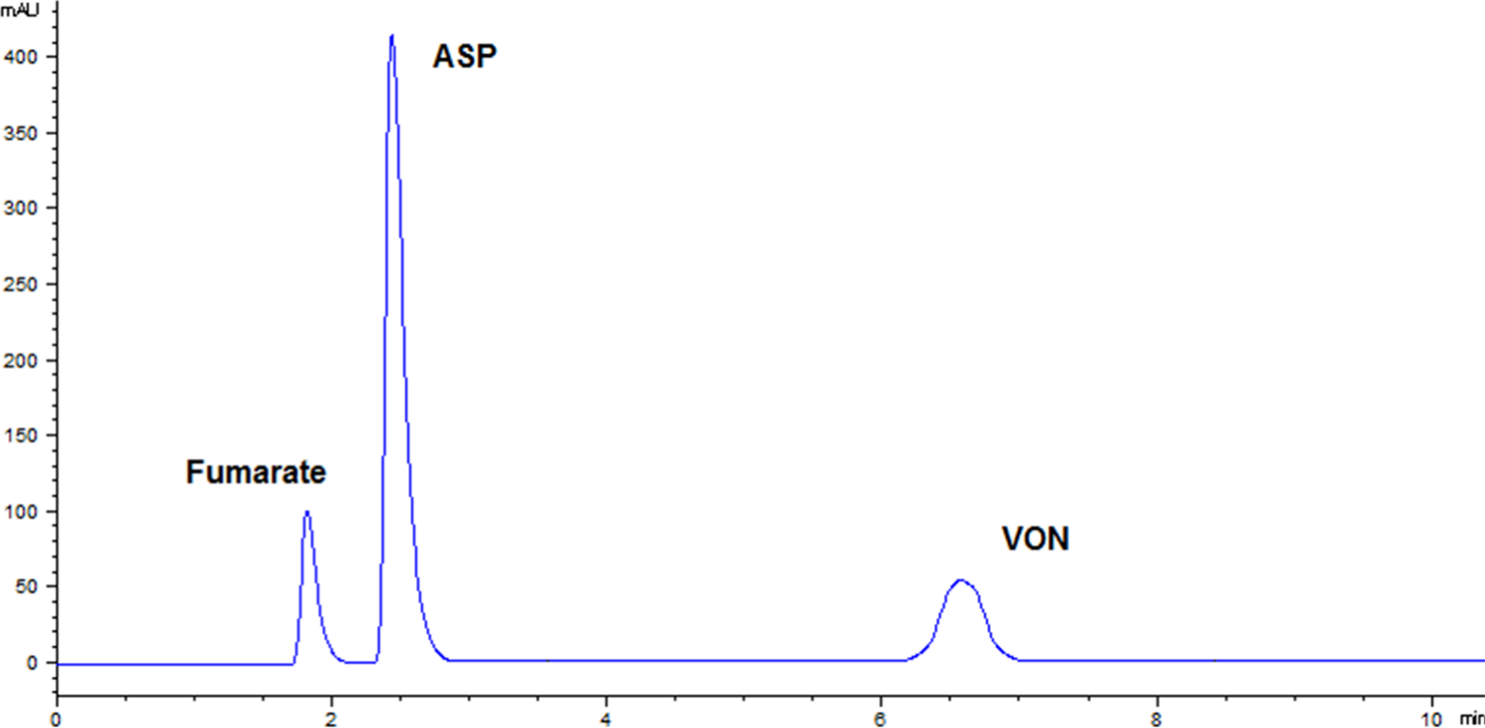
HPLC-DAD chromatogram showing VON and ASP as 10 and 100 µg/mL, respectively in lab-prepared tablets in their dosage form ratio using injection volume of 30 µL at 230 nm

Concerning the mobile phase, both acetonitrile and methanol being organic phases were tried but acetonitrile gave lower pressure and smoother baseline. Also, different aqueous phases were tried but when acetonitrile was added initially to water acidified with orthophosphoric acid at different pH values, ASP peak suffered from peak splitting and VON peak was very broad. Thus, phosphate buffer was then tried which gave better results, so different pH values of it were also tried. It was noticed that acidic pHs in the range of 3–4 caused the 2 drugs’ peaks to be tailed and distorted. However, the chosen pH buffer of 6.8 which was previously reported for VON [10] caused an improvement in the VON as well as ASP peaks. It was still noticed that VON peak was a little distorted and asymmetrical when the sample was prepared and injected in methanol or pure water. Thus, different solvents were tried to prepare the sample and the best solution which gave symmetrical peaks was to use a solvent of 50:50 water to acetonitrile. This solvent gave acceptable VON peak shape and symmetry. Using the chromatographic conditions optimized with detection at 230 nm, the two drugs showed symmetric peaks at retention times (R_t_) of 2.50 ± 0.02 and 6.59 ± 0.05 min for ASP and VON, respectively (Fig. 2). The peak observed at R_t_ of 1.79 is attributed to the fumarate ion attached to VON, as previously reported [10], did not cause interference in the assay and it did not affect the separation between the two main peaks of the two drugs which are separated with enough retention time of 4 min. Any efforts that were made to further separate the ASP peak from the fumarate peak caused further delay for VON peak which caused the run time to be longer. Longer run time consumes more solvent and jeopardizes the greenness of the assay. Thus, since the fumarate peak did not interfere in the separation of the two main peaks which was further confirmed with the system suitability parameters calculated and presented later, the chosen ratio of the mobile phase was considered the optimum one to efficiently separate the two drugs with a total run time not longer than 8 min without having to use a gradient system.

**Fig. 3:**
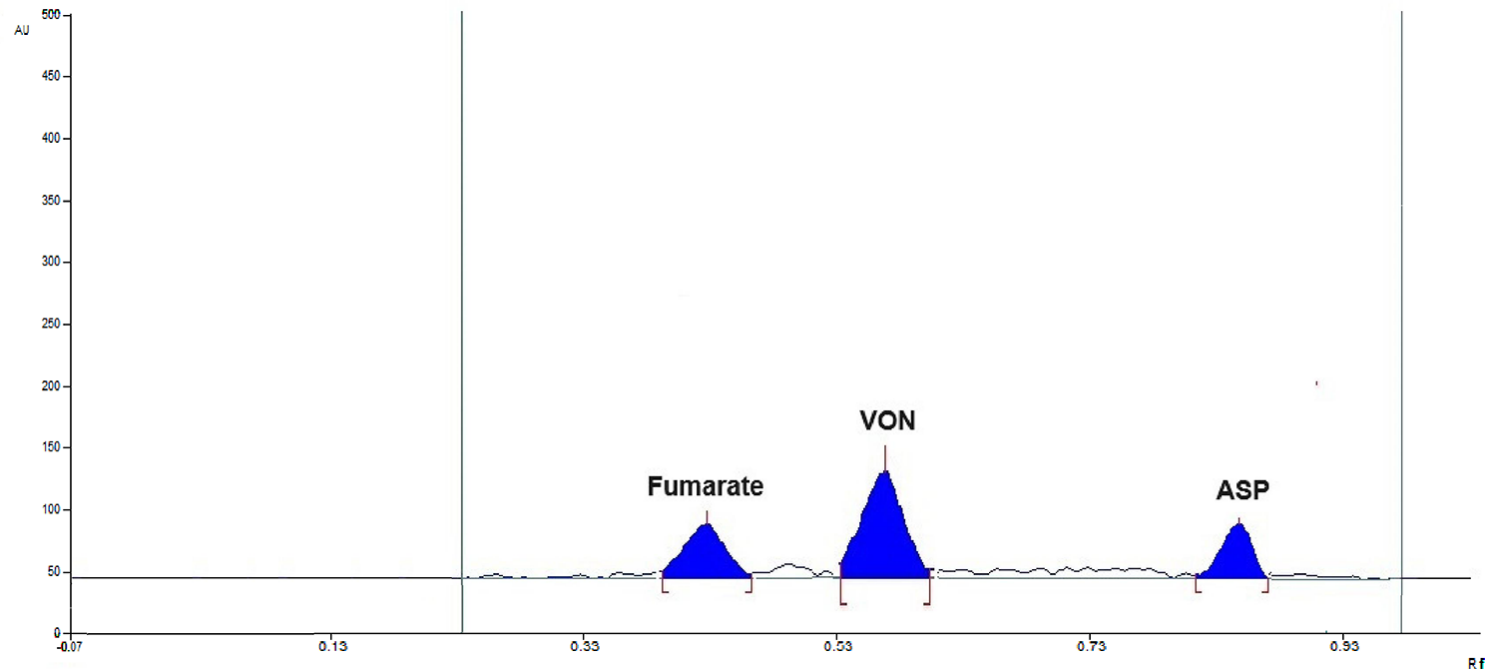
HPTLC chromatogram showing VON and ASP as 0.1 and 10 µg/band, respectively in lab-prepared tablets in their dosage form ratio using injection volume of 10 µL at 230 nm

***HPTLC method***: Different solvents with several compositions at different ratios were assessed such as methanol: water, ethyl acetate: water, methanol only, ethyl acetate: ethanol with and without ammonia. The two drugs showed symmetrical spots with good separation only with ethyl acetate: ethanol in the ratio of 5:5 and a small volume of ammonia was necessary to avoid VON peak broadening. Finally, optimum separation with minimum tailing was achieved between VON and ASP with retention factor (R_f_) of 0.54 ± 0.05 and 0.83 ± 0.05, respectively. Figure 3 shows the optimum separation of both drugs and the small of peak of fumarate also appeared at R_f_ of 0.40. Different wavelengths were tried for detection and 230 nm was chosen as optimum for simultaneous quantitation of the two drugs within the pharmaceutical preparation ratio.

### Validation

All validation parameters are tested according to International Council for Harmonization (ICH) guidelines “Q2(R1)” [20].

#### Linearity and limit of detection (LOD) / quantitation (LOQ)

The linearity of the proposed methods was assessed by analyzing the calibration standards of each of VON and ASP and plotting peak area values versus concentrations. The slopes, intercepts, correlation coefficients (r), standard deviation of residuals (Sy/x) and Standard deviations of intercept (Sa) and of slope (Sb) are given in Table 1. The regression lines of the two methods are shown in the figures of supplemteary data 1 (Additional file 1) to justify the slopes and intercepts values that might be of large value in the HPTLC as commonly known and reported previously for this technique [21, 22]. The acceptable values of the correlation coefficients of 0.999 together with the high F-values indicate the good linearity of the calibration graphs. LOD and LOQ are considered, according to the ICH, the concentrations which have a signal-to-noise ratio of 3:1 and 10:1, respectively. The LOD and LOQ for each compound were experimentally estimated and the values are presented in Table 1. The low values of LOD and LOQ ensures the proposed chromatographic methods are sensitive enough to assay VON and ASP in their combined tablets.

#### Accuracy and precision

For assessing the HPLC and HPTLC methods’ repeatability and intermediate precision, five replicates of five different synthetic mixtures of VON and ASP in different ratios were analyzed using the two proposed chromatographic methods including the dosage form ratio. Repeatability assessment, known as intra-day or intra assay precision, is concerned with the results’ precision repeated under similar experimental conditions within short time interval. Thus, the assay was repeated five times (*n* = 5) in the same day on the five different synthetic mixtures. Meanwhile, for assessing the methods’ intermediate precision which is assessing the results closeness within expected laboratories variations, known as inter-day precision, the replicate assay was repeated on five different days. All percentage relative error E_r_ (%) values and calculated percentage relative standard deviation % RSD were found to be less than 2% as reported [20] indicating good accuracy and precision of the proposed methods, respectively (Table 2).

#### Robustness

It is of great importance to ensure that the methods of assay are capable of tolerating external un- avoidable and un-detectable factors such as deviations in wavelength scales, pH adjustments and mobile phase ratios. The target was to make small changes in influensive parameters to monitor if the HPLC and the HPTLC method will be affected regarding the recoveries of the drugs or the separation efficacy (changes in resolution (R_t_/R_f_)) and also to determine the extent by which the parameters can be changed (level of tolerance). According to the ICH, the degree by which different parameters can be changed without influencing the method’s results is defined as “Robustness”. The two proposed chromatographic methods’ robustness was evaluated by the assay of VON and ASP synthetic mixture in three replicates (*n* = 3) each time changing a method parameter is made, this is followed by calculating the mean recoveries of the three runs and their RSD%. The parameters studied are demonstrated in Table 3 and changing these parameters caused no change in the determination of both drugs as shown by low values of % RSD (less than 2%) of peak areas and retardation factor (R_f_) which ensures no influence was made upon these changes. The results demonstrated in Table 3 proves that changing the mentioned parameters within the limits mentioned, will not affect the method’s results and will still be valid.

#### Selectivity

Selectivity is defined as the method’s ability to assay a specific chemical entity in prescence of other chemically related substances. The selectivity was validated by using the chromatographic methods on synthetic mixtures prepared with different ratios of VON and ASP, where good recovery and deviation results (Table 2) indicates no interference. Application of the methods for determination of VON and ASP in presence of expected dosage form’s excipients; without interference also demonstrate their selectivity (Table 4).

**Fig. 4:**
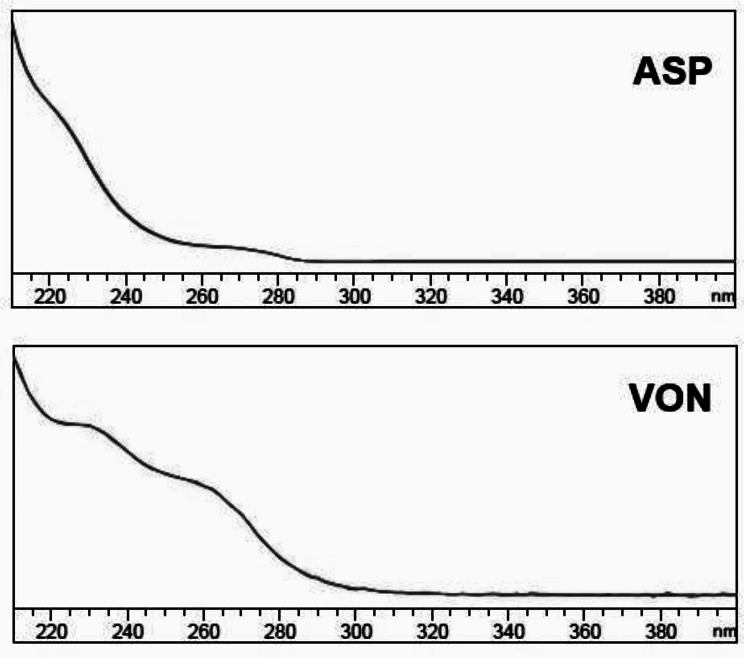
Overlaid UV spectra illustrating peak purity of VON and ASP obtained from their lab-prepared tablets for the HPLC method

The HPLC peak purity of VON and ASP was also checked and the purity angles were within the purity threshold limits. In addition, spectra of VON and ASP were recorded at different points across their peaks and their overlap indicates purity of peaks in presence of dosage form excipients (Fig. 4).

For the HPTLC method, the spots for both drugs in their samples (with excipients) were assured by comparing both R_f_ and spectra of the spot against standards. The HPTLC peaks purity was evaluated also by comparison of the spectra at positions across each peak: peak start-S, peak apex-M and peak end-E. The calculated r (S, M) and r (M, E) values were higher than 0.999 indicating the homogeneity of the peaks.

### System suitability parameters

All calculations of System suitability parameters were done and gave satisfactory method performance data (Table 5). The calculated values were compared to the reference values: capacity factor k′ (2 − 10), Selectivity α > 1, Resolution R_s_ > 2, Symmetry factor A_f_ (0.8–1.2) and Column efficiency plates.m^− 1^ (> 2000) and all results were within the mentioned limits. This shows that the HPLC method proposed is valid and achieves the enough required separation between the two drugs with acceptable peak shapes and without interference.

**Table 1:** Regression parameters for determination of VON and ASP in their dosage form

Parameter	HPLC		HPTLC	
	VON	ASP	VON	ASP
Linearity range	0.5–10 µg/mL	1–100 µg/mL	0.1–2 µg/band	0.1–10 µg/band
LOQ	0.5 µg/mL	1 µg/mL	0.1 µg/band	0.1 µg/band
LOD	0.17 µg/mL	0.33 µg/mL	0.03 µg/band	0.03 µg/band
Intercept, (a)	-43.29	-44.58	944.69	703.56
Slope, (b)	76.48	50.44	2098.15	84.07
Correlation coefficient, (r)	0.9991	0.9998	0.9993	0.9992
Standard deviation of intercept, S_a_	9.00	28.86	54.26	9.99
Standard deviation of slope, S_b_	1.83	0.59	44.27	1.99
Standard deviation of residuals, S_y/x_	14.16	46.39	67.26	16.84
F	1749.79	7391.92	2246.06	1788.46
Significance F	3.01 × 10^− 5^	3.47 × 10^− 6^	2.07 × 10^− 5^	2.91 × 10^− 5^

**Table 2:** Precision and accuracy assessment

**HPLC method**		**Accuracy and Intra-day precision,** (***n*** **=** **5**)				**Accuracy and Inter-day precision, **(***n*** **=** **5**)			
**Concentration (µg/mL)**		**VON**		**ASP**		**VON**		**ASP**	
**VON**	**ASP**	**Mean % Recovery± %RSD**	**%E**_r_	**Mean % Recovery± %RSD**	**%E**_r_	**Mean % Recovery± %RSD**	**%E**_r_	**Mean % Recovery± %RSD**	**%E**_r_
4	40	99.98 ± 0.58	-0.02	100.35 ± 0.55	0.35	101.22 ± 0.90	1.22	100.50 ± 1.33	0.50
10	10	100.55 ± 0.66	0.55	101.25 ± 0.60	1.25	99.50 ± 0.88	-0.50	101.45 ± 0.98	1.45
0.5	100	100.50 ± 0.80	0.50	99.95 ± 0.89	-0.05	99.66 ± 0.75	-0.34	100.99 ± 1.05	0.99
10	1	100.99 ± 0.95	0.99	101.52 ± 0.97	1.52	100.21 ± 1.04	0.21	100.65 ± 0.55	0.65
10	100	99.20 ± 0.72	-0.80	98.89 ± 0.66	-1.11	100.98 ± 1.21	0.98	99.99 ± 0.25	-0.01
**HPTLC method**		**Accuracy and Intra-day precision,** (***n*** **=** **5**)				**Accuracy and Inter-day precision, **(***n*** **=** **5**)			
**Concentration (µg/band)**		**VON**		**ASP**		**VON**		**ASP**	
**VON**	**ASP**	**Mean % Recovery± %RSD**	**%E**_r_	**Mean % Recovery± %RSD**	**%E**_r_	**Mean % Recovery± %RSD**	**%E**_r_	**Mean % Recovery± %RSD**	**%E**_r_
1	10	101.98 ± 1.32	1.98	100.55 ± 1.50	0.55	100.99 ± 0.90	0.99	99.95 ± 0.85	-0.05
0.1	0.1	101.56 ± 1.59	1.56	101.35 ± 1.99	1.35	101.65 ± 1.05	1.65	100.57 ± 1.22	0.57
1	0.1	100.97 ± 1.63	0.97	100.98 ± 0.98	0.98	99.97 ± 1.50	-0.03	101.55 ± 1.58	1.55
0.1	10	98.35 ± 0.90	-1.65	99.25 ± 1.00	-0.75	98.50 ± 1.55	-1.50	100.20 ± 1.70	0.20
0.5	1	98.10 ± 1.05	-1.90	98.98 ± 0.85	-1.02	99.65 ± 1.25	-0.35	99.09 ± 0.80	-0.91

**Table 3:** Robustness assessment of the proposed chromatographic methods

Parameters tested	HPTLC method				Parameters tested	HPLC method			
	VON		ASP			VON		ASP	
	RSD % peak areas	*R*_f_ ± SD	RSD % peak areas	*R*_f_ ± SD		RSD % peak areas	*R*_t_ ± SD	RSD % peak areas	*R*_t_ ± SD
1) **Mobile phase composition** [Ethyl acetate: ethanol: Ammonia (5:5:0.05, 5.5:4.5:0.05, 4.5:5.5:0.05, 5:5:0.1 (v/v))]	1.04	0.54 ± 3.85 × 10^–2^	0.85	0.83 ± 2.05 × 10^–2^	1) **Mobile phase ratio** [± 0.5% aqueous phase]	0.99	6.60 ± 4.04 × 10^–2^	1.20	2.51 ± 1.64 × 10^–2^
2) **Mobile phase volume** [15, 20 and 25 mL]	0.89	0.54 ± 1.00 × 10^–2^	0.99	0.83 ± 5.48 × 10^–3^	2) **Flow rate** [1 ± 0.05 mL/min]	0.89	6.60 ± 5.22 × 10^–2^	1.22	2.50 ± 1.34 × 10^–2^
3) **Duration of saturation** [30, 40 and 50 min]	0.94	0.55 ± 8.95 × 10^–3^	1.20	0.84 ± 1.10 × 10^–2^	3) **Column temp.** [25^o^ C ± 5^o^ C]	1.20	6.59 ± 1.14 × 10^–2^	1.17	2.50 ± 4.47 × 10^–3^
4) **Time from chromatography to scan** [10, 20, 30 and 60 min]	1.25	0.57 ± 4.47 × 10^–2^	1.24	0.84 ± 8.94 × 10^–3^	4) **pH of the aqueous phase** [6.8 ± 0.2]	1.55	6.57 ± 2.74 × 10^–2^	0.89	2.51 ± 1.41 × 10^–2^
5) λ **(± 2 nm)**	1.01	0.54 ± 8.37 × 10^–3^	0.99	0.84 ± 1.79 × 10^–2^	5) λ **(± 2 nm)**	1.01	6.60 ± 2.17 × 10^–2^	1.50	2.50 ± 8.37 × 10^–3^

**Table 4:** Application in laboratory prepared dosage form

Lab-prepared labs	% Found ± RSD % (*n* = 5)					
	VON			ASP		
	HPLC method	HPTLC method	Reported spectrophotometric method (6)	HPLC method	HPTLC method	Reported spectrophotometric method (6)
	100.45 ± 0.91	100.14 ± 1.01	100.27 ± 0.86	101.10 ± 0.66	99.32 ± 0.61	100.25 ± 1.06
Students’ *t- test*(*t*)^*^	0.76	0.83	--------	0.17	0.13	--------
Variance ratio F- test (F)^*^	1.11	1.00	--------	2.55	3.24	--------

*Theoretical values of *t* and *F*: 2.31 & 6.39, respectively, at 95% confidence limit

**Table 5:** System suitability testing of the chromatographic HPLC peaks

Analyte	Retention time (*R*_t_), min	Capacity factor (k′)	Selectivity (α)	Resolution (*R*_s_)	Asymmetry (A)	Efficiency (plates/m)
Fumarate	1.85	2.08	1.52	4.51	0.99	3181.24
ASP	2.50	3.17	3.15	12.49	0.98	4056.96
VON	6.59	9.98	------	------	0.84	2802.89

### Analysis of pharmaceutical preparations

Since the combined tablets are still not available in our markets, the methods were tested for determination of both drugs in presence of ingredients commonly present in tablets to mimic the marketed tablets. All recovery and RSD % results were acceptable (Table 4) indicate the methods are selective for VON and ASP in presence of tablet’s excipients and can be used for routine quality control of these tablets. The two proposed chromatographic methods were statistically compared to the reported spectrophotometric one [6] using *t*- test and F-test and the results showed no significant difference between the methods.

The proposed HPLC and HPTLC methods were also compared to the two reported methods (Supplementary data 2 in Additional file 1). The proposed chromatographic HPLC method was of higher sensitivity compared with the spectrophotometric reported one. Meanwhile, the spectrofluorimetric method was of better sensitivity but involves reaction of VON with derivatizing agent to give it a fluorescent property. The reaction involves pH adjustments, optimization of reaction conditions and several steps in comparison to the HPLC and HPTLC methods proposed which are direct and does not require any sample preparation. Since green analytical chemistry approaches require minimizing the analytical steps, this makes the proposed direct HPLC and HPTLC methods of advantage despite their higher linearity ranges which is still sensitive enough to determine the two drugs in their co-formulated tablets.

### Greenness and whiteness sustainability assessment

Recently, there has been consensus on the importance of regularly evaluating the eco-friendliness and effectiveness of published analytical methods using various metrics and tools to ensure sustainability and ecological compatibility. Among the latest tools for assessing eco-friendliness are AGREE (Analytical GREEnness Metric Approach) [23] and ComplexMoGAPI (Complementary Modified Green Analytical Procedure Index) [24]. Additionally, the sustainability and validity of an analytical method can be verified through the whiteness assessment using the new algorithm RGB 12 model (Red-Green-Blue) [25]. Both methods were compared in terms of their eco-friendliness and effectiveness using AGREE, ComplexGAPI, and the RGB 12 model.

**Fig. 5:**
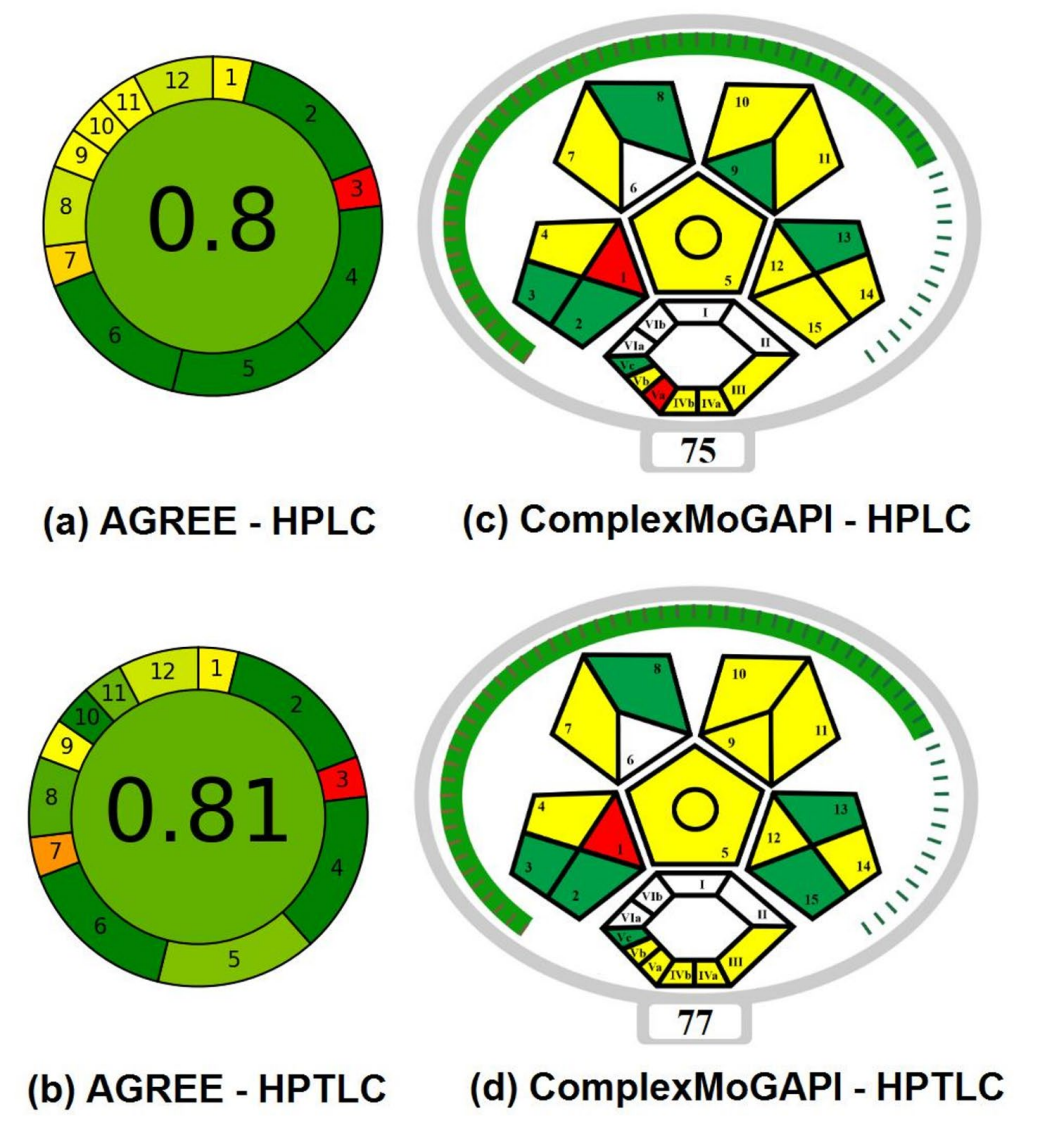
Greenness assessment using AGREE for HPTLC method & HPLC method and ComplexMoGAPI for HPTLC method & HPLC method

Commencing with the AGREE system, it evaluates greenness through an easy approach, encompassing 12 fundamental principles of GAC (green analytical chemistry). Utilizing user-friendly data entry software, it generates results represented in a clock-like pictogram format, displaying a greenness score at the center, ranging from 0 to 1. A score of 1 is depicted in dark green, while 0 is shown in dark red. Comparative analysis of two proposed methods reveals that the HPTLC method boasts a slightly higher greenness score of 0.81, attributed to its reduced energy consumption and employment of less toxic solvents compared to the HPLC method, which scored 0.80. Both methods generally achieve high greenness scores, evidenced by the red coloration in quadrant 3 of the pictogram, indicative of offline analysis (Fig. 5a and b).

In the other hand, the ComplexMoGAPI is the development of the GAPI tool [26]. It allows to assess more information and then provide a more comprehensive evaluation. This was done by adding an additional hexagonal glyph related to the processes done before the analysis on the original GAPI 5 pentagrams and giving a score which is out of 100 to evaluate the total greenness of the method. As shown in Fig. 5c and d, the HPTLC method with score 77 has more green compartments and less red compartments than that of the HPLC method with score 75 and this is attributed to the same 2 factors mentioned in the AGREE assessment (less energy and less toxic solvents). Also both methods share the same red compartment in the first pentagram due to the offline analysis. Overall the ComplexMoGAPI scores for both HPLC and HPTLC methods prove the greenness of both methods.

**Fig. 6:**
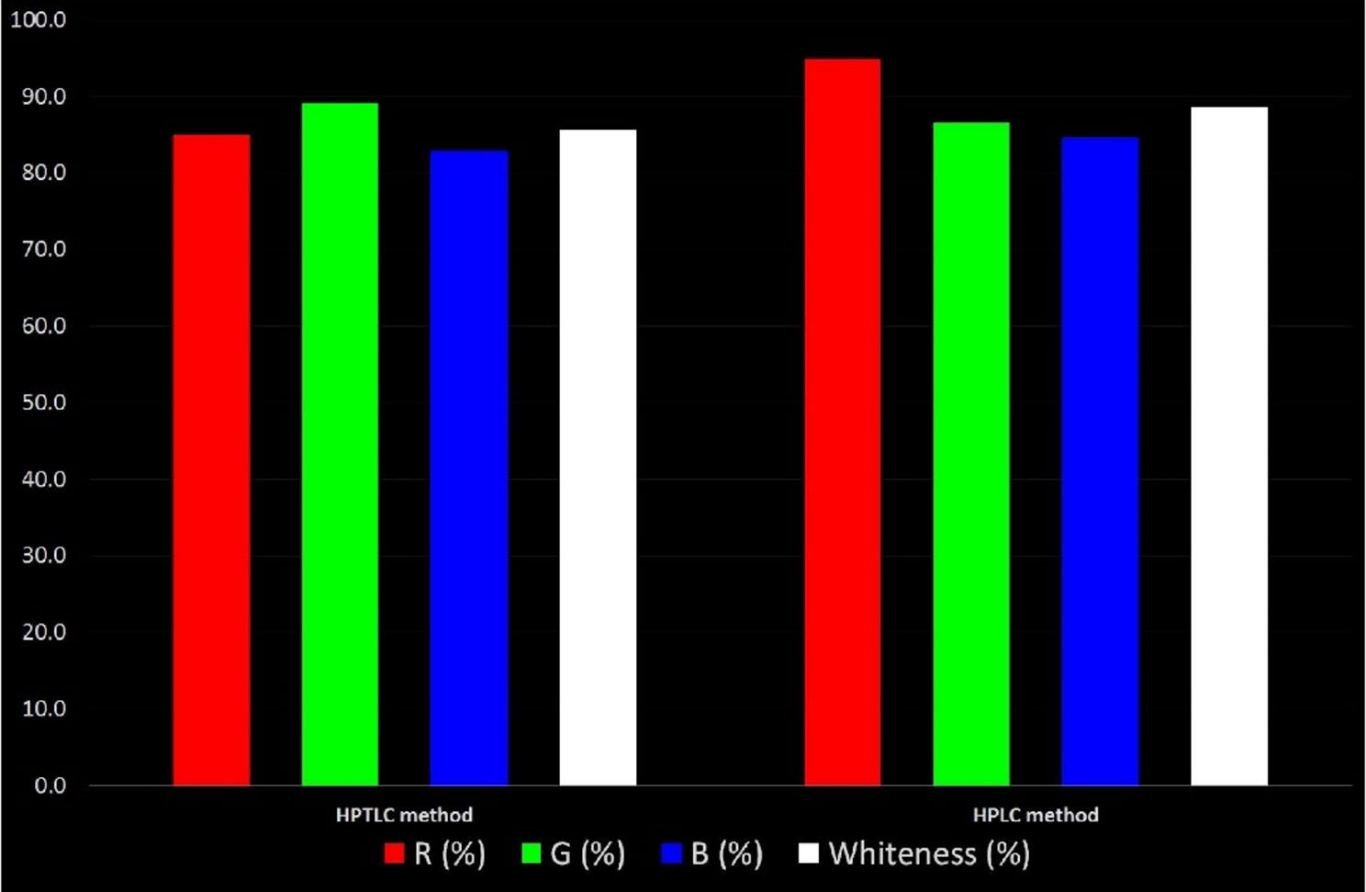
Whiteness assessment of both HPTLC and HPLC methods using the RGB 12 model

Moreover, a novel “Red Green Blue (RGB) 12” model has recently emerged in the literature for assessing “whiteness”. This model divides the assessment into three zones, each characterized by a distinct color and containing specific parameters of the analytical procedure. It evaluates the method’s greenness, represented by the green zones corresponding to GAC principles, it also assesses the validation of the analytical results in the red zones, and the sustainability and productivity of the method in the blue zones. A comparison of the two proposed methods for their whiteness is illustrated in Fig. 6. The findings from the green zone aligned closely with the greenness evaluations conducted using the AGREE and ComplexGAPI. Specifically, the HPTLC method attained a greenness score of 89.2%, while the HPLC method scored 86.7%. In contrast, the red zone assessments focusing on validation performance favored the HPLC method, attributed to its outstanding % recoveries, %RSD, and minimal detection and quantitation limits. Concerning the blue zone, optimal productivity was observed with the HPLC method, primarily due to its rapid analysis, reduced sample consumption, and high degree of automation. Combining the results from all three areas, the HPLC method emerged as the top performer with an overall whiteness score of 88.8%, while the HPTLC method scored 85.7%.These scores prove the whiteness of both methods and their high sustainability, productivity and greenness.

Assessing the two proposed methods with the aforementioned assessment matrices, proves the environmental sustainability of these methods and that these methods comply with the goal of the 12 principles for green and white analysis. The obtained results show that the methods are not only green but also valid and by this it achieved the target that should be complied in the analytical field by substituting the current available techniques with greener ones without jeopardizing the validity of the results [25, 27]. Detailed data is supplied in supplementary data 3 in Additional file 1.

## Conclusion

The proposed HPLC and HPTLC methods are the first chromatographic methods to be reported for this newly marketed formulation of VON and ASP. The proposed methods are simple and accurate so they could be useful for routine quality control analysis of the concerned drugs either in their bulk form or in combined drug products. The two proposed chromatographic methods have the advantage over the previously reported ones in using small amounts of solvents and reagents, minimum steps with no sample preparation and short analysis time. In addition, both methods were proved to be green and white methods and can be used in routine analysis.

## Electronic supplementary material

Below is the link to the electronic supplementary material.

Supplementary Material 1

## Data Availability

All data generated or analysed during this study are included in this published article and its supplementary information files.

## Abbreviations

NSAID: Non-Steroidal Anti-Inflammatory Drug
COX: Cyclooxygenase
CABG: Coronary Artery Bypass Graft Surgery
AFib: Atrial Fibrillation
PPI: Proton Pump Inhibitors
VON: Vonoprazan Fumarate
ASP: Aspirin
FDCs: Fixed Dose Drugs Combination
ICH: International Council for Harmonization
R_t_: Retention Times
R_f_: Retention Factor
DAD: Diode Array Detection
LOD: Limit of Detection
LOQ: Limit of Quantitation
E_r_ (%): Percentage Relative Error Values
% RSD: Percentage Relative Standard Deviation
AGREE: Analytical GREEnness Metric Approach
ComplexMoGAPI: Complementary Modified Green Analytical Procedure Index
RGB 12 model: Red-Green-Blue 12 model
GAC: Green Analytical Chemistry
