# Supplementary material for: Chromatographic assay of recently approved co-formulation of Vonoprazan fumarate with low dose Aspirin: AGREE, Complex MoGAPI, and RGB 12-model assessments
Source: BMC Chem. 2024 Nov 16;18(1):230. doi: 10.1186/s13065-024-01344-7 (PMC11568669; doi:10.1186/s13065-024-01344-7)
Supplement: Supplementary file 1 — Supplementary Material 1 [file 13065_2024_1344_MOESM1_ESM.docx]

**Supplementary data 1: Calibration curves for VON and ASP using the HPTLC methods.**

**Supplementary data 2: Comparison of the proposed methods with the reported ones.**

| Method of analysis | | Mobile phase /solvent used | Stationary phase | Detector | λ (nm) of detection | Retention times / retention factors | Linearity range | Application |
| --- | --- | --- | --- | --- | --- | --- | --- | --- |
| Spectrophotometry [6] | | ASP and VON stock solutions  were prepared in 10 mL  DMSO and 40 mL ethanol. Further dilutions were made using ethanol. | --------- | --------.  --------. | Ratio difference: ASP was quantified using differences in amplitudes 229 and 283 nm, and  VON was quantified using the differences in amplitudes 255 and 212 nm.  First derivative ratio spectra: ASP determined at 237.40 nm and VON determined at 244 nm. | --------. | ASP and VON linear over ranges of 2–25 and 1–10 µg/mL, respectively. | Applied for determination of VON and ASP in synthetic mixtures and tablets. |
| Spectrofluorimetry [9] | | --------- | --------- | ---------  --------- | ASP λ_exc_ = 295 nm and λ_em_ = 405 nm. Upon derivatization of VON with NBD-Cl, the product was quantified at λexc = 465 nm and λem = 537 nm. | --------. | ASP and VON linear over ranges of 2.0 – 100.0 [ng.mL](http://ng.ml/)^−1^ and 5.0-200.0 [ng.mL](http://ng.ml/)^−1^. | Pharmaceutical analysis of both drugs. |
| Proposed methods | HPLC | Phosphate buffer of pH 6.8 and acetonitrile in ratio of 63:37 with flow rate of 1 mL.min^−1^ | Reversed phase C18 column (250×4.6 mm, 5 μm) | Diode Array Detector | 230 nm for detection of both drugs | 2.50 ± 0.02 and 6.59 ± 0.05 min for ASP and VON, respectively | ASP and VON linear over ranges of 1-100 and 0.5-10 µg/mL, respectively. | Applied for determination of VON and ASP in synthetic mixtures and laboratory prepared tablets. |
|  | HPTLC | ethyl acetate : ethanol (75 %) : ammonia (5:5:0.05) | 20x10 cm aluminum plates [silica gel-60 _(F254)_], from E. Merck, Germany. | Densitometric scanning using deuterium lamp and Camag scanner-III |  | 0.83 ± 0.05 and 0.54 ± 0.05, for ASP and VON, respectively | ASP and VON linear over ranges of 0.1-2 and 0.1-10 µg/band, respectively. |  |

| **RED PRINCIPLES (analytical performance)** |  |  | **R1: Scope of application** | **R2: LOD and LOQ** | **R3: Precision** | **R4: Accuracy** |
| --- | --- | --- | --- | --- | --- | --- |
|  | **Method number** | **Method name** | 0-100 | 0-100 | 0-100 | 0-100 |
|  | **1** | **HPTLC method** | 80 | 80 | 90 | 90 |
|  | **2** | **HPLC method** | 90 | 90 | 100 | 100 |

**Supplementary data 3: RGB 12 whiteness assessment method tables**

| **BLUE PRINCIPLES (practical side)** |  |  | **B1: Cost-efficiency** | **B2: Time-efficiency** | **B3: Requirements** | | **B4: Operational simplicity** | | |
| --- | --- | --- | --- | --- | --- | --- | --- | --- | --- |
|  | **Method number** | **Method name** | 0-100 | 0-100 | Sample consumption (0-100) | Other needs: advanced instruments, skills, facilities (0-100) | Miniaturization (0-100) | Integration and automation (0-100) | Portability (0-100) |
|  | **1** | **HPTLC method** | 90 | 80 | 80 | 90 | 80 | 80 | 70 |
|  | **2** | **HPLC method** | 80 | 90 | 90 | 80 | 90 | 90 | 70 |

| **GREEN PRINCIPLES (green chemistry)** |  |  | **G1: Toxicity of reagents (impact and biodegradation)** | **G2: Amount of reagents and waste** | **G3: Concumption of energy and other media** | **G4: Direct impacts (safety, use of animals and GMOs)** | | |
| --- | --- | --- | --- | --- | --- | --- | --- | --- |
|  | **Method number** | **Method name** | 0-100 | 0-100 | 1-100 | Safety of users (0-100) | Use of animals (0 if no, 1 if yes) | Use of GMO (0 if no, 1 if yes) |
|  | **1** | **HPTLC method** | 90 | 80 | 90 | 90 | 0 | 0 |
|  | **2** | **HPLC method** | 80 | 90 | 80 | 90 | 0 | 0 |

| **Method number** | **Method name** | **R (%)** | **G (%)** | **B (%)** | **Whiteness (%)** |
| --- | --- | --- | --- | --- | --- |
| **1** | **HPTLC method** | 85.0 | 89.2 | 82.9 | 85.7 |
| **2** | **HPLC method** | 95.0 | 86.7 | 84.6 | 88.8 |

| **Method: HPTLC method** | | | | | |
| --- | --- | --- | --- | --- | --- |
| R1: Scope of application | 80.0 | G1: Toxicity of reagents | 90.0 | B1: Cost-efficiency | 90.0 |
| R2: LOD and LOQ | 80.0 | G2: Amount of reagents and waste | 80.0 | B2: Time-efficiency | 80.0 |
| R3: Precision | 90.0 | G3: Energy and other media | 90.0 | B3: Requirements | 85.0 |
| R4: Accuracy | 90.0 | G4: Direct impacts | 96.7 | B4: Operational simplicity | 76.7 |
| **85.0** | | **89.2** | | **82.9** | |
| **85.7** | | | | | |

| **Method: HPLC method** | | | | | |
| --- | --- | --- | --- | --- | --- |
| R1: Scope of application | 90.0 | G1: Toxicity of reagents | 80.0 | B1: Cost-efficiency | 80.0 |
| R2: LOD and LOQ | 90.0 | G2: Amount of reagents and waste | 90.0 | B2: Time-efficiency | 90.0 |
| R3: Precision | 100.0 | G3: Energy and other media | 80.0 | B3: Requirements | 85.0 |
| R4: Accuracy | 100.0 | G4: Direct impacts | 96.7 | B4: Operational simplicity | 83.3 |
| **95.0** | | **86.7** | | **84.6** | |
| **88.8** | | | | | |
